# Supplementary material for: Divergent wiring of repressive and active chromatin interactions between mouse embryonic and trophoblast lineages
Source: Nat Commun. 2018 Oct 10;9:4189. doi: 10.1038/s41467-018-06666-4 (PMC6180096; doi:10.1038/s41467-018-06666-4)
Supplement: Supplementary file 1 — Supplementary Information [file 41467_2018_6666_MOESM1_ESM.pdf]

Divergent wiring of repressive and active chromatin interactions between mouse embryonic and trophoblast lineages

Schoenfelder, Mifsud, et al.

Supplementary Figures

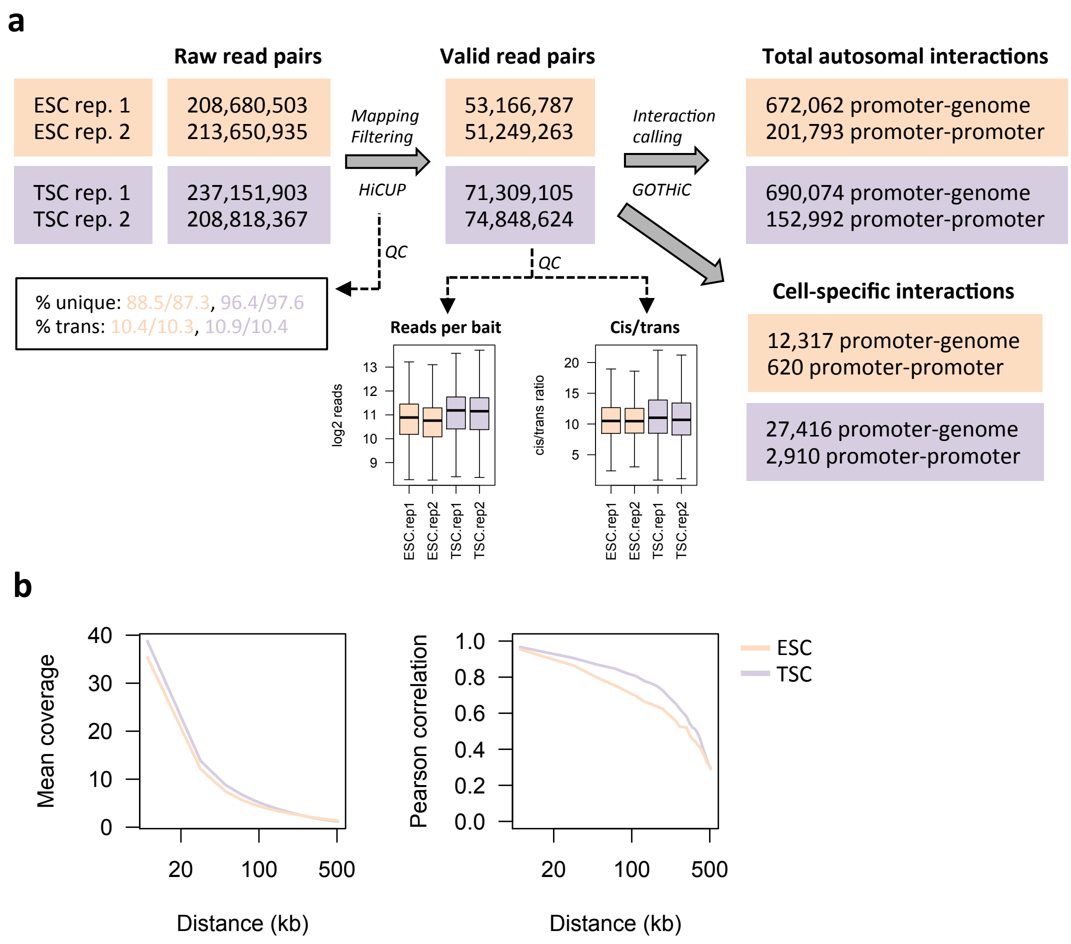

Supplementary Figure 1 – a) Read counts and quality control metrics for the PCHi-C runs. Valid read pairs do not include duplicated reads. Boxplots show the number of reads (log2) per captured promoter bait, and the ratio between *cis* and *trans*-interacting fragments per promoter. For interaction calling, read numbers were matched between ESC and TSC and reads from sex chromosomes were excluded. Cell-specific interactions were called using a modified version of GOTHiC (see Methods). b) Mean coverage and correlation coefficient between both replicates as a function of distance, at single restriction fragment resolution. All correlations displayed are highly significant (p<2.2E-16).

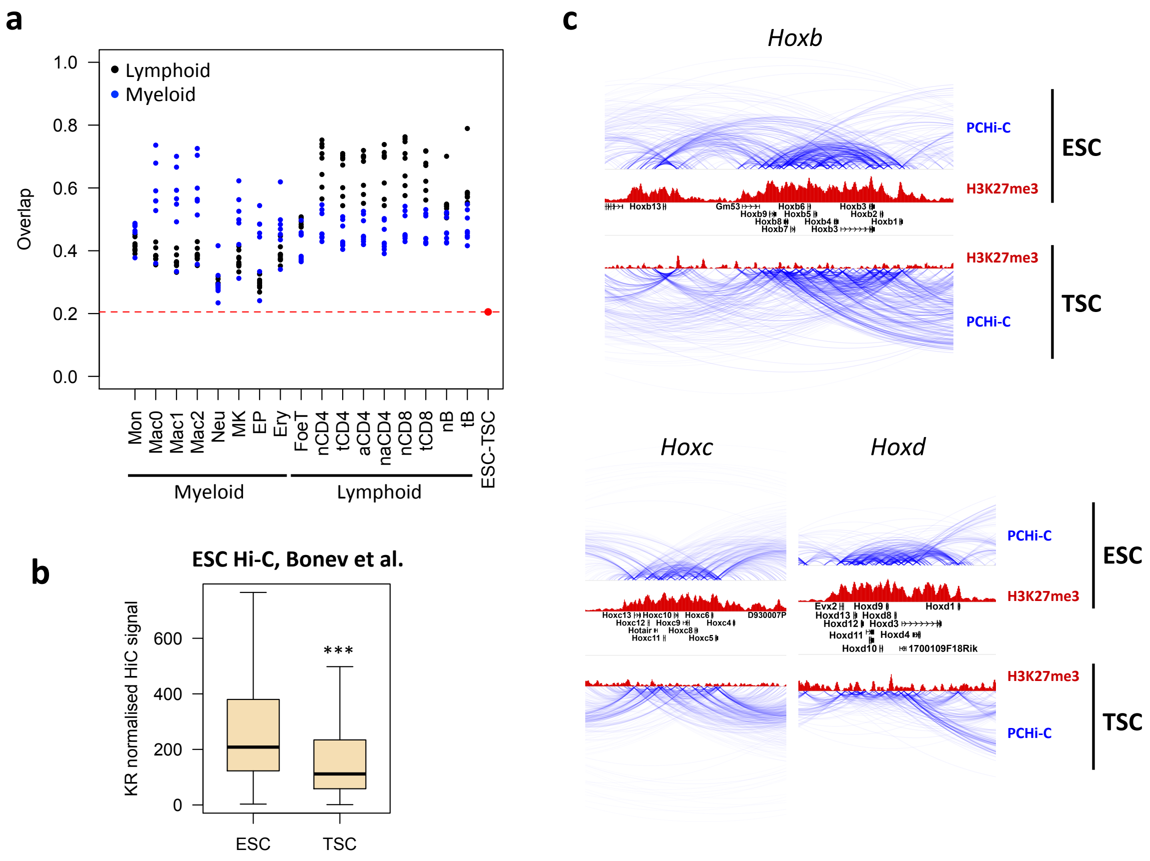

Supplementary Figure 2 – a) Pairwise comparison between interactions for PCHi-C data from multiple blood cell types. For each cell type on the x axis, the fraction of overlap with all other cell types is plotted. The overlap between ESCs and TSCs is plotted separately (in red). b) ESC-specific interactions display higher signal than TSC-specific interactions in an independent Hi-C dataset from a different ESC line[31]. Knight-Ruiz normalised data are displayed, at 5kb resolution. Boxplot midline represents median, box edges the first and third quartiles, and whisker edges are the last data points within 1.5x the interquartile range. ***p<2.2E-16 (Wilcoxon test). c) Genome browser views of the *Hoxb*, *Hoxc* and *Hoxd* clusters.

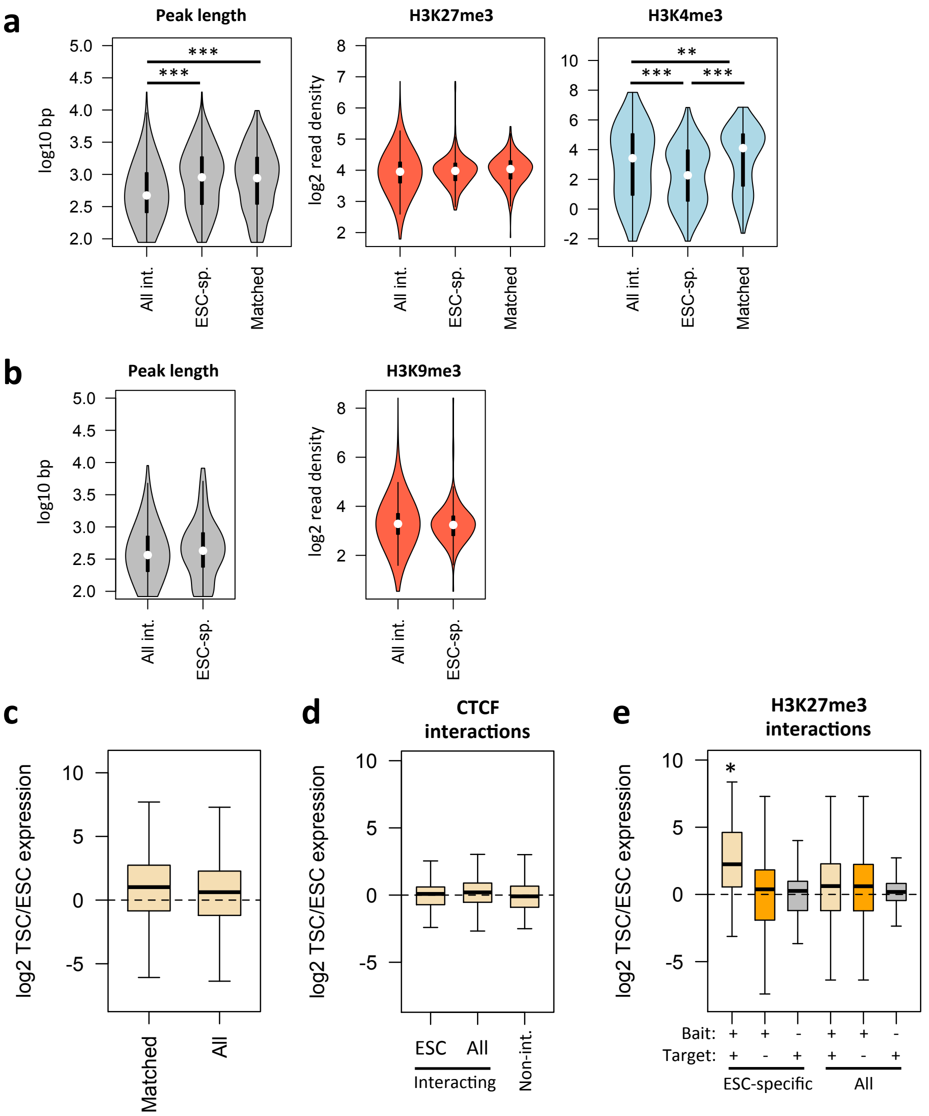

Supplementary Figure 3 – a) Peak length and ChIP-seq signal density distributions at H3K27me3 peaks associated with all H3K37me3 homotypic interactions or ESC-specific ones, or for shared interactions involving a matched set of H3K27me3 peaks. **p<0.001, ***p<1E-13 (Kolmogorov-Smirnov test, corrected for multiple comparisons). b) Peak length and ChIP-seq signal density distributions at H3K9me3 peaks associated with all H3K9me3 homotypic interactions or ESC-specific ones. c) Expression ratio (log2) between TSCs and ESCs for genes associated with the matched set of peaks defined in b). d) TSC/ESC expression ratio (log2) for genes involved in either CTCF homotypic interactions (all or ESC-specific), and compared with genes not involved in homotypic interactions. e) TSC/ESC expression ratio (log2) for genes involved in interactions with H3K27me3 at the bait, target or both ends. * p<0.05, comparing ESC-specific with all interactions (t-test, corrected for multiple comparisons). Boxplot midline represents median, box edges the first and third quartiles, and whisker edges are the last data points within 1.5x the interquartile range.

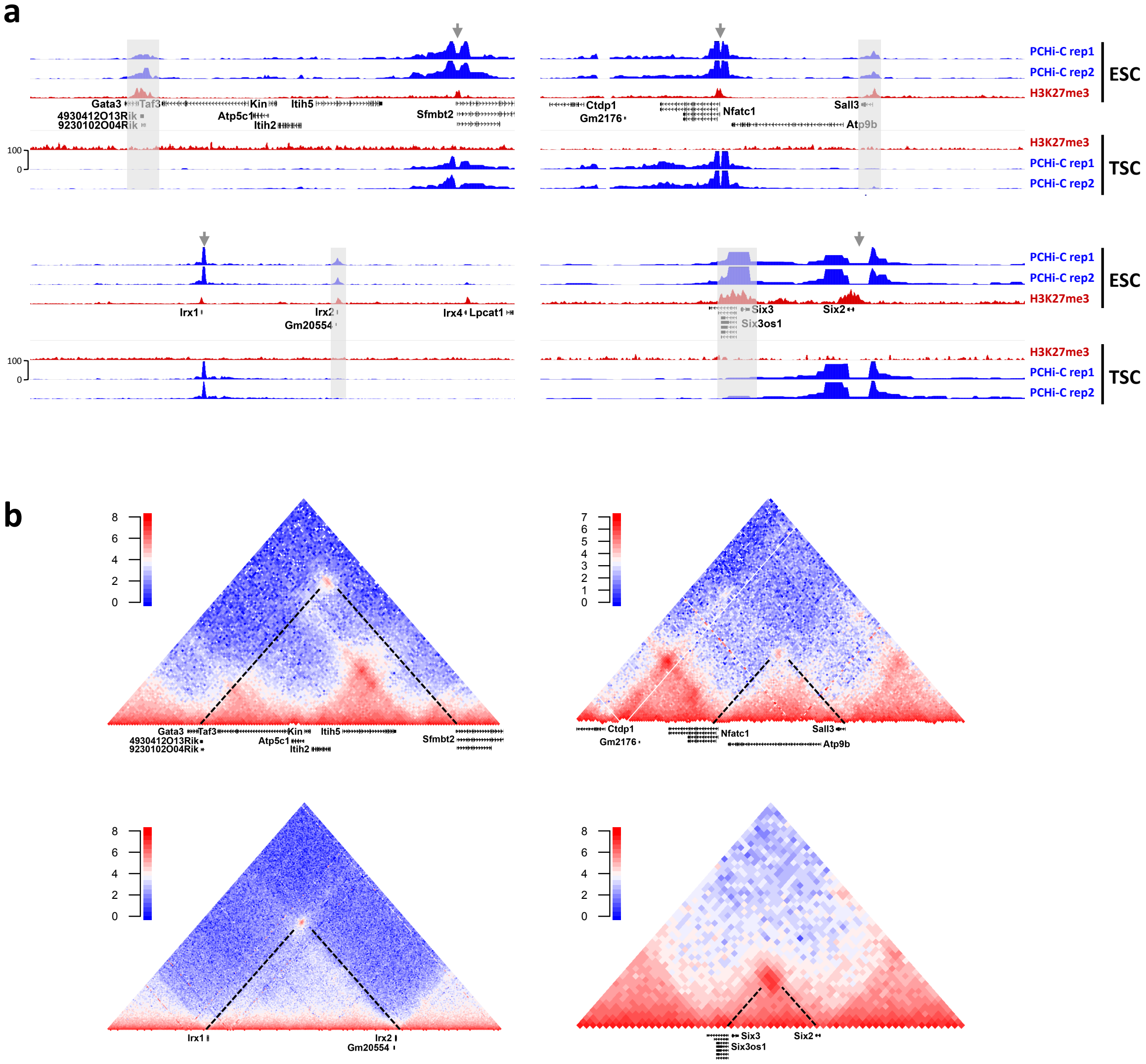

Supplementary Figure 4 – a) 4C-like tracks of H3K27me3-associated interactions, displaying read counts for each restriction fragment interacting with the indicated bait (arrow). The TSC replicate 1 track displays raw read counts, with the remaining tracks being scaled to this track. b) Hi-C heatmaps for the same regions, extracted from published genome-wide Hi-C data in ESCs[31]. Log2-transformed read counts of Knight-Ruiz normalised data are displayed, at 5kb resolution. H3K27me3-associated interactions are highlighted by the dashed lines.

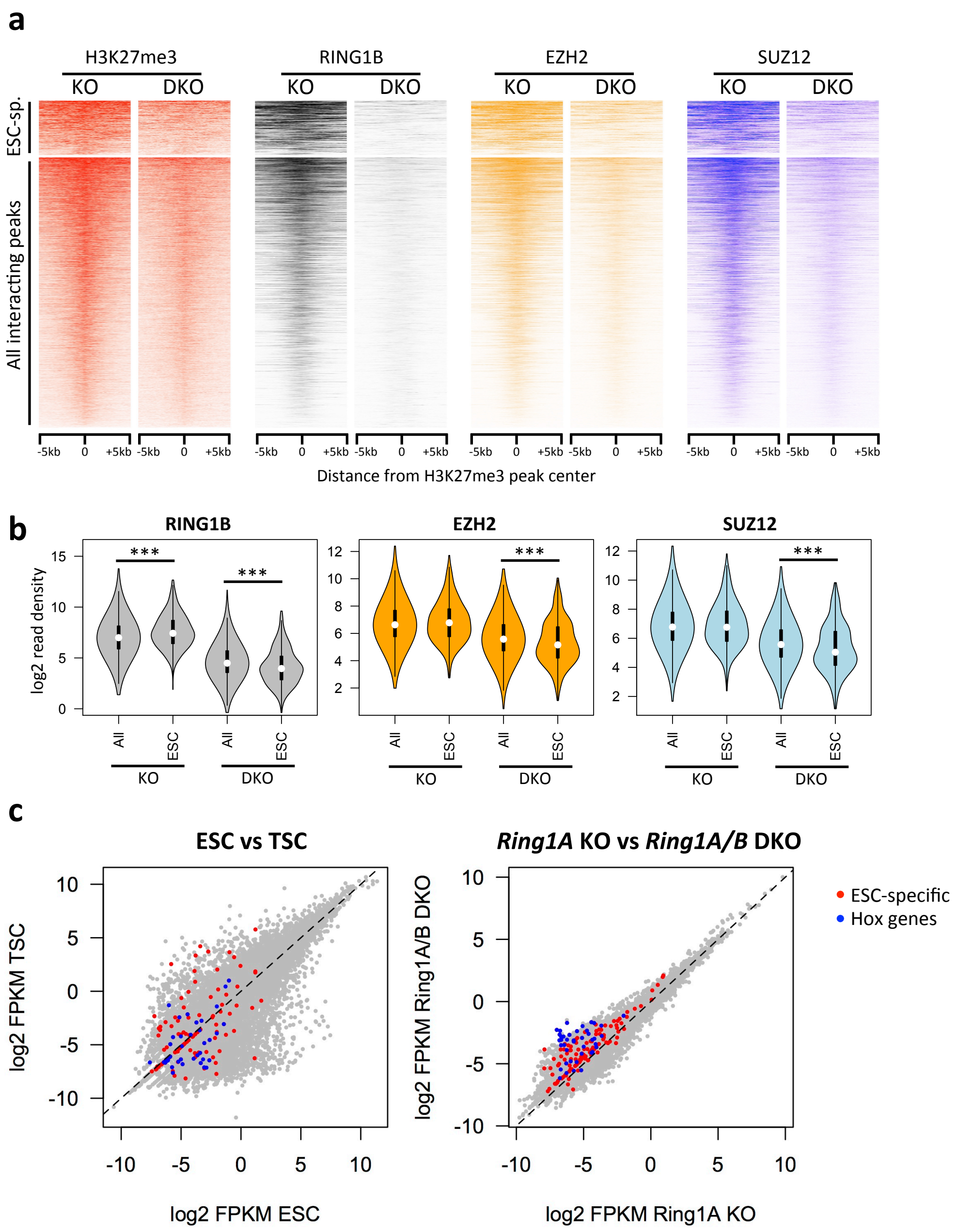

Supplementary Figure 5 – a) H3K27me3, RING1B, EZH2 and SUZ12 ChIP-seq signals in *Ring1A* KO and *Ring1A/B* DKO ESCs, centered on the peaks involved in ESC H3K27me3 homotypic interactions. Top heatmaps are for ESC-specific interactions only. b) ChIP-seq signal density distributions at H3K27me3 peaks associated with all H3K27me3 homotypic interactions or ESC-specific ones, in *Ring1A* KO and *Ring1A/B* DKO ESCs. ***p<1E-5 (Kolmogorov-Smirnov test, corrected for multiple comparisons). c) Expression of *Hox* genes (blue) and genes involved in ESC-specific H3K27me3 homotypic interactions (red).

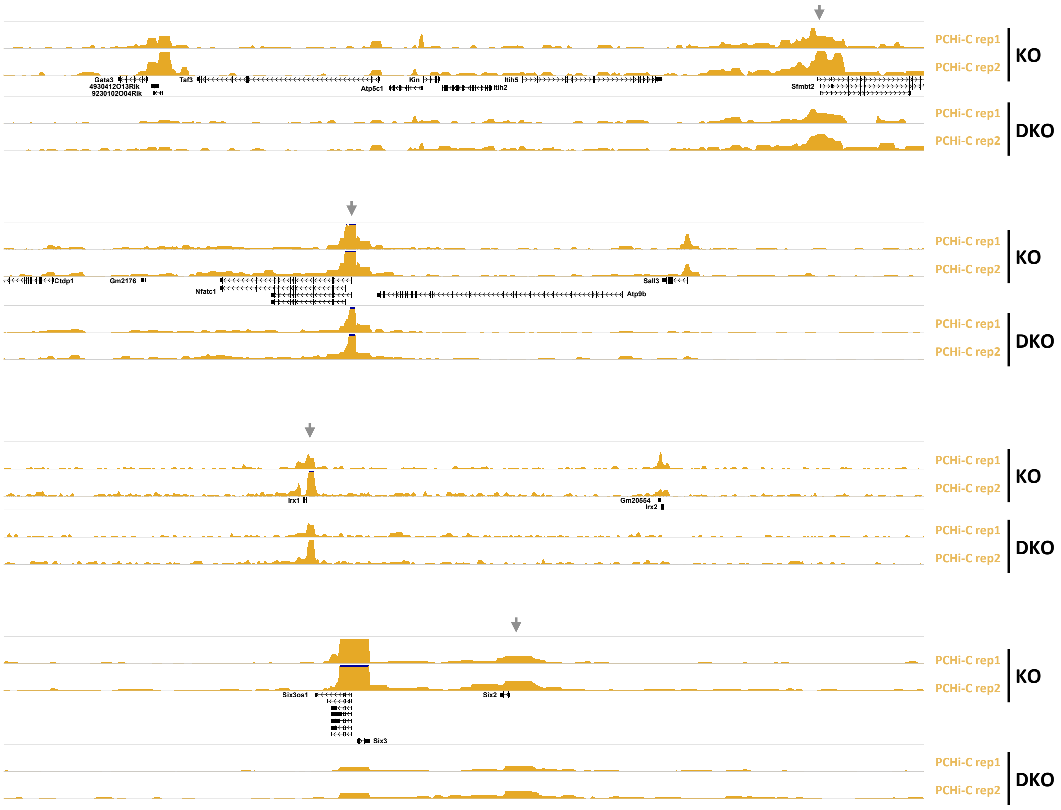

Supplementary Figure 6 – 4C-like tracks of H3K27me3-associated interactions in *Ring1A* KO and *Ring1A/B* DKO ESCs. The PCHi-C signal is displayed for each restriction fragment interacting with the indicated bait (arrow).

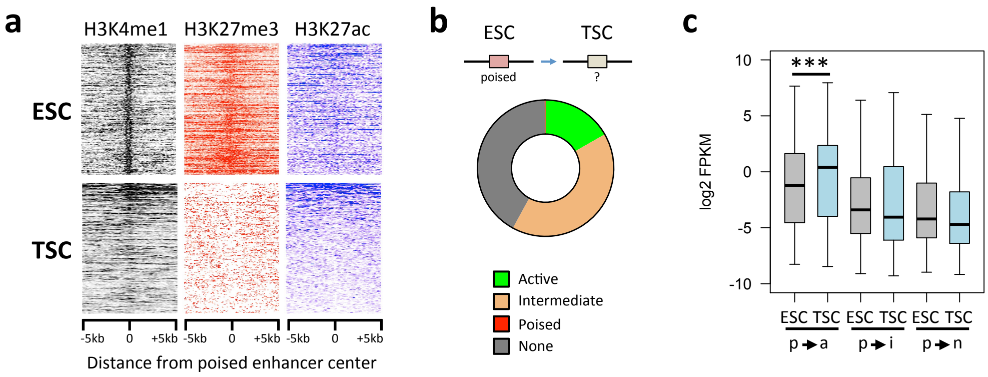

Supplementary Figure 7 – a) H3K4me1, H3K27me3 and H3K27ac ChIP-seq signals centered on ESC poised enhancers. b) Proportion of ESC poised enhancers that are absent ('none') or present in an active or intermediate state in TSCs (only 1 enhancer was in the poised state in TSCs). c) Expression of genes interacting with enhancers that are poised in ESCs but active (p->a), intermediate (p->i) or absent (p->n) in TSCs. *** p<0.0005 (paired t-test, corrected for multiple comparisons). Boxplot midline represents median, box edges the first and third quartiles, and whisker edges are the last data points within 1.5x the interquartile range.

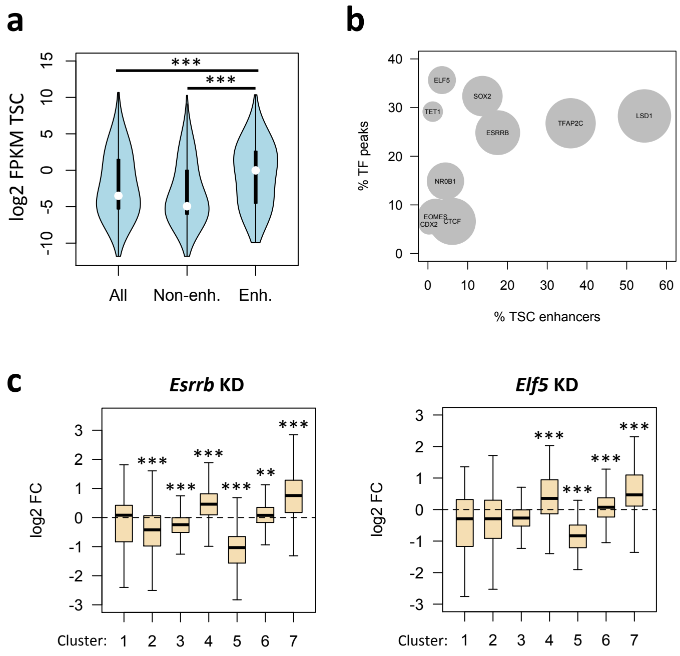

Supplementary Figure 8 – a) Expression of genes interacting with active enhancers in TSCs, compared to genes that do not interact with any active enhancer, or to all genes. ***p<2.2E-16 (Kolmogorov-Smirnov test, corrected for multiple comparisons). b) Proportion of active TSC enhancers bound by each of the indicated transcription factors, and compared with the proportion of respective ChIP-seq peaks overlapping active enhancers. The circle size is proportional to the number of ChIP-seq peaks detected for each transcription factor. c) Expression fold change (log2) upon *Esrrb* or *Elf5* knockdown in TSCs for genes interacting with enhancers bound by the respective transcription factor (ESRRB or ELF5) in each expression cluster described in Figure 4d. ** p<0.001, *** p<1E-7, compared to all genes (ANOVA with Tukey post-hoc test). Boxplot midline represents median, box edges the first and third quartiles, and whisker edges are the last data points within 1.5x the interquartile range.

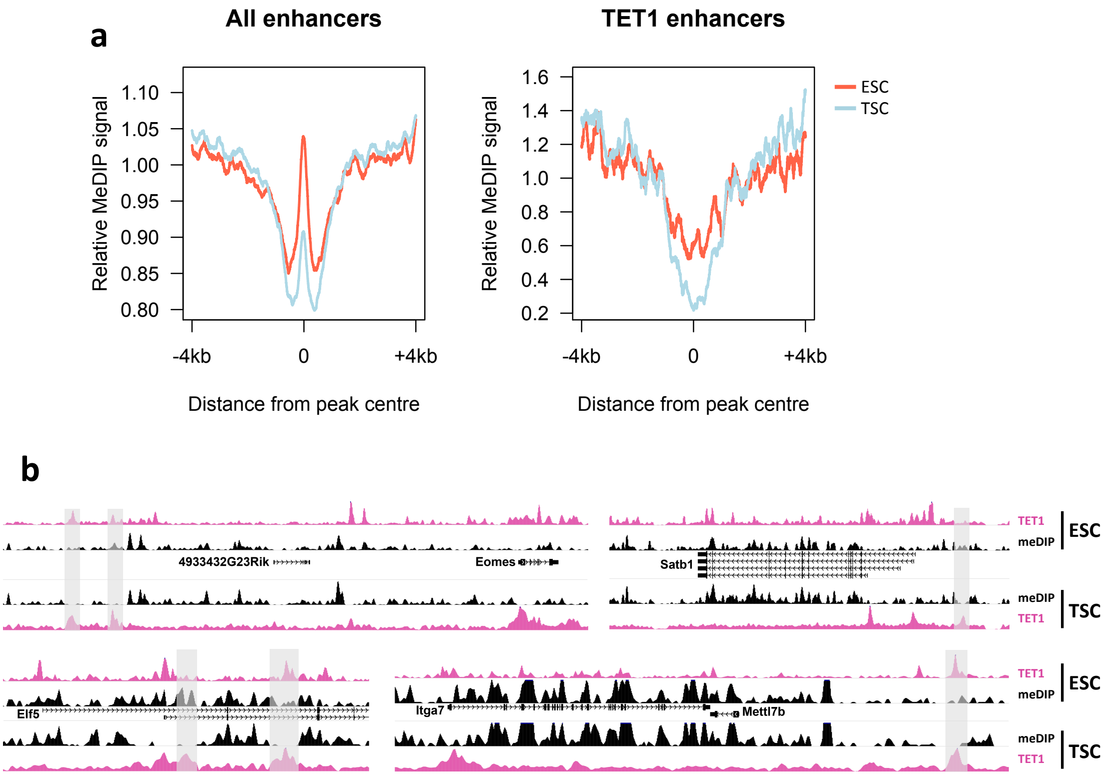

Supplementary Figure 9 – a) Genome-wide average profile of methylated DNA immunoprecipitation (meDIP) signal across all TSC enhancers (left) or TET1-bound TSC enhancers (right). Raw data from Senner et al.[23]. b) Genome browser examples of TET1-bound enhancers (highlighted by grey boxes) that interact with TSC-expressed genes, showing higher meDIP signal in ESCs compared to TSCs.
