# Supplementary material for: Divergent wiring of repressive and active chromatin interactions between mouse embryonic and trophoblast lineages
Source: Nat Commun. 2018 Oct 10;9:4189. doi: 10.1038/s41467-018-06666-4 (PMC6180096; doi:10.1038/s41467-018-06666-4)
Supplement: Supplementary file 3 — Description of Additional Supplementary Files [file 41467_2018_6666_MOESM3_ESM.pdf]

Description of Additional Supplementary Files

File Name: Supplementary Data 1
Description: List of external datasets used
